# IgE-mediated food allergy throughout life

**DOI:** 10.3906/sag-2006-95

**Published:** 2021-02-26

**Authors:** Ebru ÇELEBİOĞLU, Ayşegül AKARSU, Ümit Murat ŞAHİNER

**Affiliations:** 1 Department of Allergy and Clinical Immunology, Faculty of Medicine, Hacettepe University, Ankara Turkey; 2 Department of Pediatric Allergy and Asthma, Faculty of Medicine, Hacettepe University, Ankara Turkey

**Keywords:** Alpha-gal allergy, anaphylaxis, food allergy, food challenge, IgE, pollen food allergy, skin prick test

## Abstract

Food allergy (FA) has become an increasing problem throughout the world. Over the last 2 decades, the frequency of FA has increased in both children and adults. The prevalence differs according to the research methodology, age, and geographic regions, ranging between 2.0% and 10.0%. The most common form of FA is immunoglobulin E (IgE)-mediated FA. In this form, patients may present with life-threatening conditions, such as anaphylaxis, or milder conditions, such as urticaria, angioedema, sneezing, and nausea alone. The gold standard in the diagnosis of FA is oral provocation tests. Epidermal skin prick tests and specific IgE measurements, as well as component-resolved diagnostic techniques are helpful in the diagnosis and follow-up of patients. In this review, the epidemiology, diagnosis, follow-up, and prognosis of IgE-mediated FA in children and adults were discussed and some specific forms of FA, such as pollen FA syndrome, alpha-gal allergy, and food-dependent exercise-induced anaphylaxis were explained.

## 1. Introduction

Food allergy (FA) is defined as an adverse health effect arising from a specific immune response that occurs reproducibly on exposure to a given food [1]. It should be differentiated from nonimmune-mediated adverse food reactions, which include metabolic (e.g., lactose intolerance), pharmacologic (e.g., caffeine) and toxic (e.g., food poisoning) events [2]. FA is classified according to the type of immune response, as immunoglobulin E (IgE)-mediated, non-IgE–mediated, or mixed. The underlying mechanisms, clinical findings, diagnostic methods, management, and prognosis are different for each type [3]. This review focused on IgE-mediated FA. 

**Figure 1:**
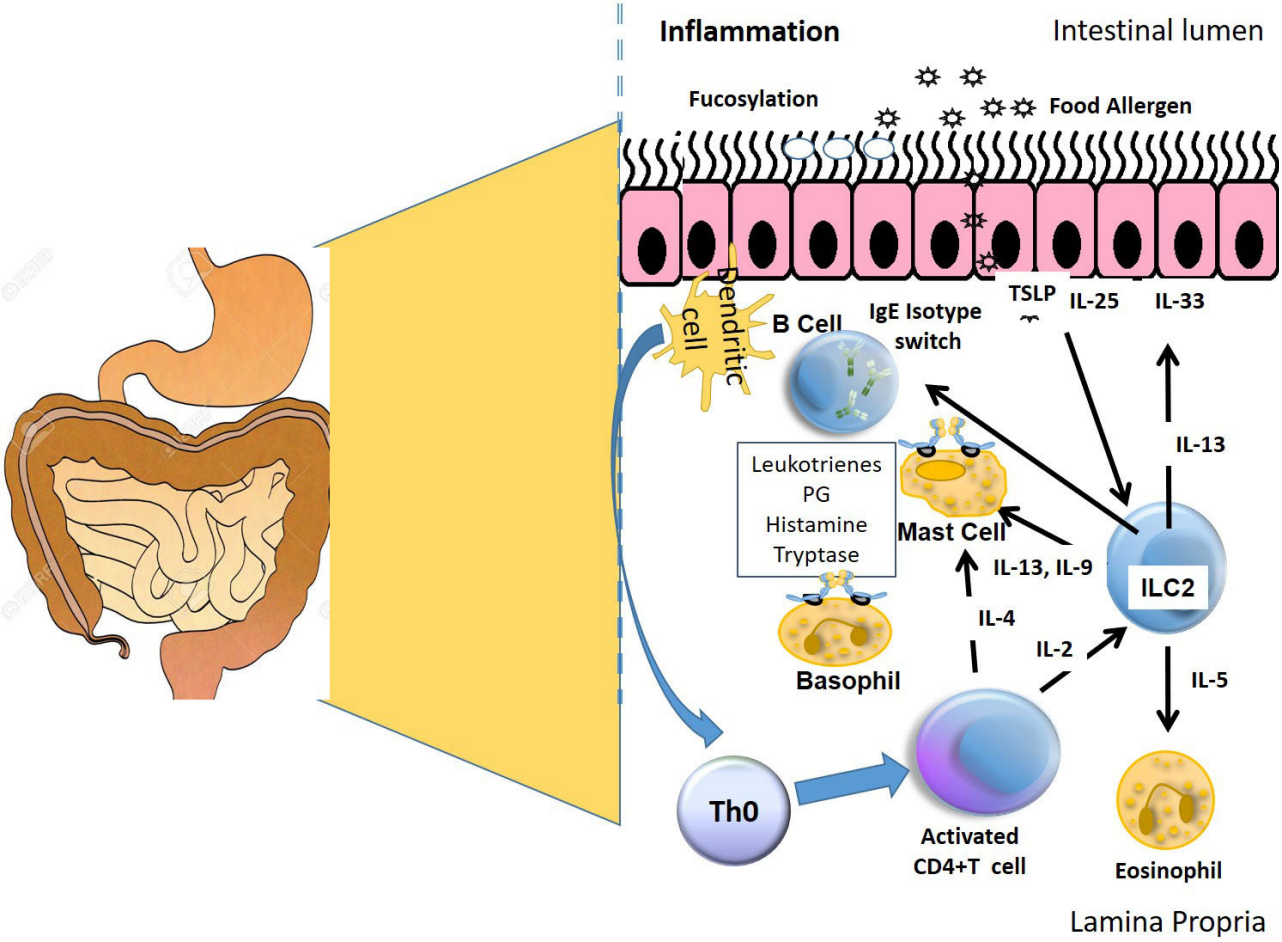
Simplified pathophysiology of IgE-mediated FA.

The pathophysiology of IgE-mediated FA is simplified in Figure 1. 

## 2. Epidemiology in food allergy

### 2.1. In children

The prevalence of FA varies between 2% and 10%, based on variants such as age, and geographical or race differences, and the description of the allergy, i.e. whether it was oral food challenge (OFC)-proven or self-reported by the patients [1]. In the study of Osborne et al. [4], 1-year-old infants were screened for sensitization to common allergens (peanuts, raw egg whites, and sesame) and sensitized infants underwent OFC. More than 10% of the 1-year-old infants were reported as allergic to at least one of the common allergens. Their follow-up study evaluated the same participants at the age of 4 years old, wherein they reported a decrease in the prevalence of challenge-proven FA as 3.8%, due to the high resolution rate of egg allergy [5]. The study of Sasaki et al. [6] reported the prevalence of clinically-defined and self-reported FA in early adolescence (10 to 14 years old) as 4.5% and 5.5%, respectively. The most common allergen was peanuts, followed by tree nuts. In the study of Chen et al. [7], 12–36-month-old toddlers were evaluated for FA and the prevalence was reported as 2.5%, wherein the most prevalent allergens were eggs, peanuts, cow’s milk, and fish. Another study from China showed that the prevalence of challenge-proven FA in 0–12-month-old infants was 3.8%, wherein the most common allergen was eggs (2.5%), followed by cow’s milk (1.3%). On the other hand, the prevalence of FA was higher when the studies included patients with self-reported FA, rather than challenge-proven. Gupta et al. reported that 11.4% of parents considered that their children had FA. As the parent-reported reaction history was not consistent with IgE-mediated FA, they excluded 4% of the children. The prevalence of FA in children was estimated as 7.6% in the USA, and the most common allergens were peanuts, milk, shellfish, and tree nuts [8]. A questionnaire-based study determined that the cumulative prevalence of FA was 6.7% in France and cow’s milk, eggs, kiwi, and peanuts were the major food allergens [9]. In Turkish children, the foods that most commonly cause FA were reported as eggs (57.8%), cow’s milk (55.9%), and hazelnuts (21.9%) [10]. Kahveci et al. showed that egg whites, cow’s milk, tree nuts, and sesame were the most prevalent allergenic foods in eastern Mediterranean children aged 0–24 months [11]. Kaya et al. showed that the OFC-proven FA prevalence in Turkish adolescents was 0.15% and the most common food allergen was peanuts, followed by tree nuts [12]. Similar prevalence was reported by Mustafayev et al.; however, walnuts were reported as the most common food causing allergic reaction [13]. Common allergens vary in the different geographic areas, possibly as the result of culinary differences. 

### 2.2. In adults

As in all other types of allergies, the incidence of FA has been increasing in both adults and children [14]. Allergy to commonly consumed foods in adults may be due to the persistence of childhood FA or it may be adult onset. The prevalence of IgE-mediated FA in adults depends on the methodology of the study, and whether the patients were included based on self-report or if a diagnostic workup was performed. Most of the FA prevalence studies have relied on self-report, International Statistical Classification of Diseases-coded1World Health Organization. International Statistical Classification of Diseases [online]. Website: https://www.who.int/classifications/classification-of-diseases [accessed 8 Feb 2021]. reactions, or the demonstration of allergen-specific IgE (sIgE), without the confirmation of symptoms with specific provocation [15]. Based on those reports, the prevalence of FA in the adult North American population was reported as 6.6%–10% [15]. However, these prevalence rates would be lower if specific provocation tests had been performed. In a metaanalysis, it was shown that there was up to a 15-fold difference between the self-reported and challenge-proven prevalence of FA, where the discordance was attributed to probable non-IgE–mediated mechanisms [16]. In another metaanalysis of the European population, the overall pooled point prevalence of symptoms with positive sIgE to at least 1 food was 2.7%, with the rate being slightly higher among children than in adults [17]. In a study of a population between 18 and 60 years of age, conducted between 1 January 2000 and 30 September 2012, an estimate of the frequency ranges of FA in Europe by self-report, positive IgE, symptoms with positive IgE, and food challenge comprised 3.5%–19.6%, 2%–21.9%, 2.2%, and 0.1%–3.2%, respectively [17]. The lifetime prevalence of FA in this age group was 9.5%–35% [17]. In a study of 774 patients with seasonal allergic rhinitis in Turkey, the prevalence of FA was the most common accompanying allergic disease (14%) [18]. In a large population-based study of 11,810 participants, the life-time prevalence of self-reported FA/nonallergic food hypersensitivity was reported as 9.5%; however, the rate was 0.1% when double-blind, placebo-controlled food challenge tests were performed [19]. As most studies are based on questionnaire data, and the prevalence of FA may be influenced by many variables, such as sex, age, nationality, and food consumption habits, the true prevalence of FA among adults is currently unknown. 

## 3. Diagnosis of food allergy

The gold standard diagnostic tool for FA is double-blind placebo-controlled OFCs (DBPCFC). Since OFC has a systemic reaction risk that includes fatal anaphylaxis, other complementary diagnostic approaches should be performed before the challenge test. Detailed clinical history, skin prick test (SPT), sIgE level, as well as component-resolved diagnostic (CRD) tests could help to define the risk of OFC [20]. Intradermal tests, atopy patch tests, allergen-specific IgG4 measurement, kinesiology, hair analysis, and electrodermal testing are not recommended for the diagnosis of FA [21–23]. 

### 3.1. Medical history

Clinical history and physical examination are the first and most important procedures for the diagnosis of FA. Although there are no specific symptoms, atopic dermatitis or clinical findings at the acute phase of the reaction might be a clue for diagnosis [1,3]. The clinical history should include suspected allergens, form of the food (baked, extensively heated, or raw), amount of the consumed food, time interval between ingestion and the reaction, outcomes of previous exposure to the same allergen before the reaction, recurrence of the IgE-mediated reaction after ingestion of the same culprit food, cofactors during the reaction (e.g., exercise, alcohol, infection, and nonsteroidal antiinflammatory drugs), treatment and the duration of the symptoms, and time of the last reaction [1,21,24]. Cutaneous (urticaria or angioedema), respiratory (rhinorrhea, sneezing, stridor, cough, wheezing, or dyspnea), gastrointestinal (nausea, vomiting, abdominal pain, or diarrhea) or cardiovascular (hypotension or shock) system symptoms within 2 h after ingestion of the culprit food, or recurrent symptoms after further exposure to the culprit food are significant clues for the diagnosis of FA [25]. Accompanying atopic diseases, including atopic dermatitis, allergic rhinitis, asthma, and eosinophilic gastrointestinal disease should also be recorded [24]. Clinical history alone is inadequate to establish a diagnosis of FA [26]. A diagnostic work-up should include SPTs, sIgE, and CRD results.

### 3.2. Skin prick test

SPT is a widely used diagnostic tool for IgE-mediated allergies because the procedure is easy to perform, reproducible, inexpensive, time-effective and highly sensitive. SPT detects allergen sIgE antibodies in vivo [27]. Allergen extracts, negative (usually 0.9% NaCl) and positive controls (histamine, 10mg/mL) are dropped to the volar surface of the forearm or upper back of the patient, separately, and they are imported into the skin layer via a 1-mm lancet or similar device. The wheal size is measured after 10–15 min. If the wheal size is 3 mm or greater than the negative control, the test result for that allergen is considered positive, in other words, the patient is sensitized to that allergen [24]. In contrast to a low-positive predictive value, SPT has a high-negative predictive value. Therefore, when the SPT result is negative, the diagnosis of FA is unlikely [26]. H1 antihistamines, long-term or high-dose systemic corticosteroids, and omalizumab should be stopped 4–5 days, 1–3 weeks, and 6 weeks, respectively, before performing the SPT to avoid false-negative results. In addition to that, topical steroids and calcineurin inhibitors could suppress the immediate skin test response when applied to the SPT area [27]. Although it is not standardized, the prick-to-prick test with fresh food can be performed when the allergen extract for the suspected food is unavailable or the SPT result using the extract is inconsistent with the clinical history [24]. For cow’s milk, eggs, and peanuts, the 95% positive predictive SPT values of clinical reactivity are described (Table 1) [28,29]. In addition, a larger wheal size is more likely to be associated with clinical reactivity to the culprit food. However, the wheal size does not indicate the severity of the reaction [30,31]. There is no international consensus on the 95% positive predictive SPT values for other foods, such as wheat, tree nuts, and fish [32].

**Table 1:** The 95% positive predictive SPT and sIgE clinical reactivity values of different foods described for children [22–24].

Allergen	Age group	95% PPV	
		sIgE kU/L	SPT (mm)
Cow’s milk	All ages ≤1 year ≤2 years ≤4 years ≤6 years	≥15 ≥5 ≥11.1 ≥11.7 ≥13.7	≥8 ≥6
Eggs	All ages ≤2 years	≥7 ≥2	≥7 ≥5
Peanuts		≥15	≥8

### 3.3. Allergen-specific IgE levels

Like SPT, positive sIgE measurement results indicate sensitivity to the culprit allergen, and results should be carefully interpreted [24]. Different test methods have been used to measure the sIgE level, each of which has advantages and disadvantages. The common feature of these systems is the in vitro determination of the allergen sIgE antibody and the results are reported in kilo units per liter (kU/L). Follow-up of sIgE levels should be checked using the same method, as the measurement differences might alter the accuracy of the results [27]. Similar to the SPT, the 95% positive predictive values of sIgE for cow’s milk, eggs, and peanuts were reported, and a high level of sIgE is a significant predictor of clinical reactivity. The predictive value of the sIgE level is affected the age of the patient (Table 1) [28–30]. 

### 3.4. Role of component-resolved diagnosis

In CRD tests, allergen specific food antigens and epitopes are detected using qualitative, semiquantitative, or quantitative assays. Therefore, high levels of sIgE due to cross-reactive components of other food antigens could be distinguished from allergenic ones [27]. Cow’s milk, eggs, peanuts, and tree nuts have well-defined components, where if positive, indicate increased likelihood of reactivity. Patients who have high levels of casein (Bos d8) and ovomucoid (Gal d1) cannot tolerate extensively heated (baked) cow’s milk and eggs, respectively. In addition, increased levels of casein and ovomucoid sIgE indicate the persistence of the FA [33,34]. Ara h 2 for peanuts, Gly m 8 for soybeans, Ana o 3 for cashews, Jug r 1 for walnuts, Cor a 9 and 14 for hazelnuts, Ses I 1 for sesame, and Fag e 3 for buckwheat have been associated with clinical FA [35–42]. Unlike SPT and sIgE results, CRD tests can predict the severity of the reaction. Ara h 2 and 6 for peanuts, and Cor a 9 and 14 for hazelnuts have been reported as risk factors for severe allergic reactions [43,44]. The cross-reactive components of the food allergens are described in Table 2. Although CRD tests are widely used, the diagnostic accuracy, cut-off values, and cost-effectiveness for FA are problems that should be addressed.

**Table 2:** Major allergen components involved in cross-reactivity between pollen and food allergens.

Allergen family	Allergen components involved in cross-reactivity
LTP (heat and digestion stable)	Ar v 3 (mugwort), Pla a 3 (London plane tree), Amb a 6 (short ragweed)Pru p 3(peach), Mal d 3 (apple), Api g 2 (celery), Sin a 3 (yellow mustard)
2S albumins	No aeroallergensAna o 3 (cashew), Ara h 2 (peanut), Pis v 1 (pistachio), Gly m 8 (soybean)
Cupin	No aeroallergensPrudu 6 (almond), Cor a 9 (hazelnut), Pis v 2 (pistachio), Ara h 1 (peanut)
PR-10 (denature with cooking/processing)	Bet v1 (birch), Que a 1 (White oak), Aln g 1 (alder), Fag s 1 (beech)Mal d 1 (apple), Pruar 1 (apricot), Api g 1 (celery), Ara h 8 (peanut)
Profilin (denature with digestion)	Bet v 2 (birch), Art v 4 (mugwort), Amba 8 (ragweed), Phl p 12 (Timothy grass)Mal d 4 (apple), Api g 4 (celery), Pru p 4 (peach), Ara h 5 (peanut)

### 3.5. Oral food challenges

OFC is the only procedure that could establish the diagnosis of FA. Since DBPCFC, which is the gold standard method for diagnosis, is a time-consuming procedure and requires a standard method of food processing, patients frequently undergo open or single-blind food challenges. DBPCFC should be performed in patients who have defined subjective and psychological symptoms in order to avoid false positive results and unnecessary food restriction [25]. In addition, OFCs for research settings should be performed with double-blind procedures [45]. OFC with suspicion of IgE-mediated allergy must be performed in an office or hospital setting, where the personnel and equipment are adequate to treat a severe allergic reaction [20]. Similar to SPT, certain medications (H1- and H2-antihistamines, atypical antidepressants/sedatives, benzodiazepines, and tricyclic antidepressants), which have a suppressant effect on the test results, should be discontinued before the procedure. The interval between the last dose of the drug and the challenge test depends on the half-life of the medication. In addition, the patient should discontinue their medications, including ACE inhibitors, beta-blockers, cromolyn, nonsteroidal antiinflammatory drugs, proton pump inhibitors, short, medium, and long-acting bronchodilators, oral bronchodilators, 5 half-lives before the OFC to avoid severe reactions [45]. In Japanese FA guidelines, low, medium, and full dose food challenge procedures were described. It was proposed that the decision for a challenge dose should be made according to the risk analysis of the patient, including their clinical history, SPT, sIgE, and CRD results. Although, a high-dose challenge should be performed for FA labeling [46], in the consensus report of Ebisawa et al., at least 2 g of food protein was suggested as the top dose to prevent false negative results. The general challenge schedule consists of half-logarithmic dose increments, from 3 mg to 3 g of food protein, in order to avoid severe reactions. Dose intervals should be at least 20 min. The challenge test should be stopped when the patient has any objective symptoms and proper treatment should be provided [20]. If the patient describes subjective symptoms, the challenge may continue until objective symptoms occur or the dose interval can be extended, the dose in the previous step can be repeated, or the test can be repeated as DBPCFC [47]. 

## 4. Prognosis of food allergy in children

Children who have cow’s milk, eggs, soy, and wheat allergies could tolerate the allergen at a rate resolution range of 52%–79%, 49%–68%, 69%, and 65%, respectively. In contrast to these allergens, peanuts, tree nuts, and fish allergies remain throughout the life span of an individual in most cases [1]. Sicherer et al. reported that 49.3% of children who have an egg allergy could tolerate eggs at a median age of 72 months [48]. Savage et al. predicted a 68% resolution rate of egg allergy at the age of 16 years [49]. Similar to eggs, allergy to cow’s milk may resolve in 52.6% of patients at a median age of 63 months, and 79% of patients could be tolerant by the age of 16 years [50,51]. Keet et al. showed that the median age of tolerance to wheat allergy was approximately 78 months and the rate of resolution was 65% by 12 years of age [52]. The study of Peters et al. [53] demonstrated that peanut allergy resolved in 22% of patients by the age of 4 years. The rate of resolution of tree nut allergy was reported as 10% in 3–21-year-old patients [54].

## 5. Treatment of food allergy

To date, no curative treatment option has been reported for FA [1]. Acute treatment includes the management of allergic reactions and anaphylaxis [55]. Elimination diets, education on allergen labeling, cross-contamination, and scheduled clinical follow-up are the main tools of management [56]. Strict avoidance of the culprit food might cause nutritional deficits. Therefore, dietary consultation should be performed, especially if the patient has multiple FAs and nutritional support should be considered in order to avoid nutritional deficiency [25]. 

Three decades ago, allergen-specific immunotherapy for FA was described. Several studies about the efficacy of oral, epicutaneous, and sublingual immunotherapies for peanuts, eggs, cow’s milk, and hazelnuts were reported [57–60]. The main aim of immunotherapy is to provide desensitization or sustained unresponsiveness to the offending allergen. Desensitization could be accomplished after months of therapy, which provides tolerance to the allergen during the treatment phase. After cessation of immunotherapy, most desensitized patients could not tolerate the allergen. However, in sustained unresponsiveness, patients could ingest the allergenic food with no clinical reaction for several months after the end of the therapy. This could be achieved only in some patients who received immunotherapy for years [56].

In oral immunotherapy (OIT), an allergen powder is ingested daily at increasing doses (initial dose escalation and dose build-up, from micrograms to 300–4000 mg protein/ day) until the maintenance phase. The initial and build-up dose escalation phases should be performed under the supervision of a physician. Maintenance doses could be self-ingested at home. This phase may take months or years [61]. OIT has higher desensitization and sustained unresponsiveness ratio than those of other immunotherapy modalities. However, OIT has the risk of serious adverse events, which include systemic reactions and the development of eosinophilic esophagitis. Gastrointestinal side-effects have been reported as the most common dose-limiting adverse reactions [62,63]. Omalizumab, a monoclonal anti-IgE antibody, can be administered during the escalation phase in order to decrease the severity of IgE-mediated adverse events [64]. More recently, the FDA approved the first drug for peanut OIT. U.S. Food & Drug Administration (2020). FDA approves first drug for treatment of peanut allergy for children [online]. Website: https://www.fda.gov/news-events/press-announcements/fda-approves-first-drug-treatment-peanut-allergy-children [accessed 22 March 2020].

In sublingual and epicutaneous immunotherapy, allergen extract drops and patches are applied, respectively, at lower doses than OIT. Compared to OIT, those methods are safer; however, the rates of desensitization and sustained unresponsiveness are lower than those of OIT [56]. 

**Figure 2:**
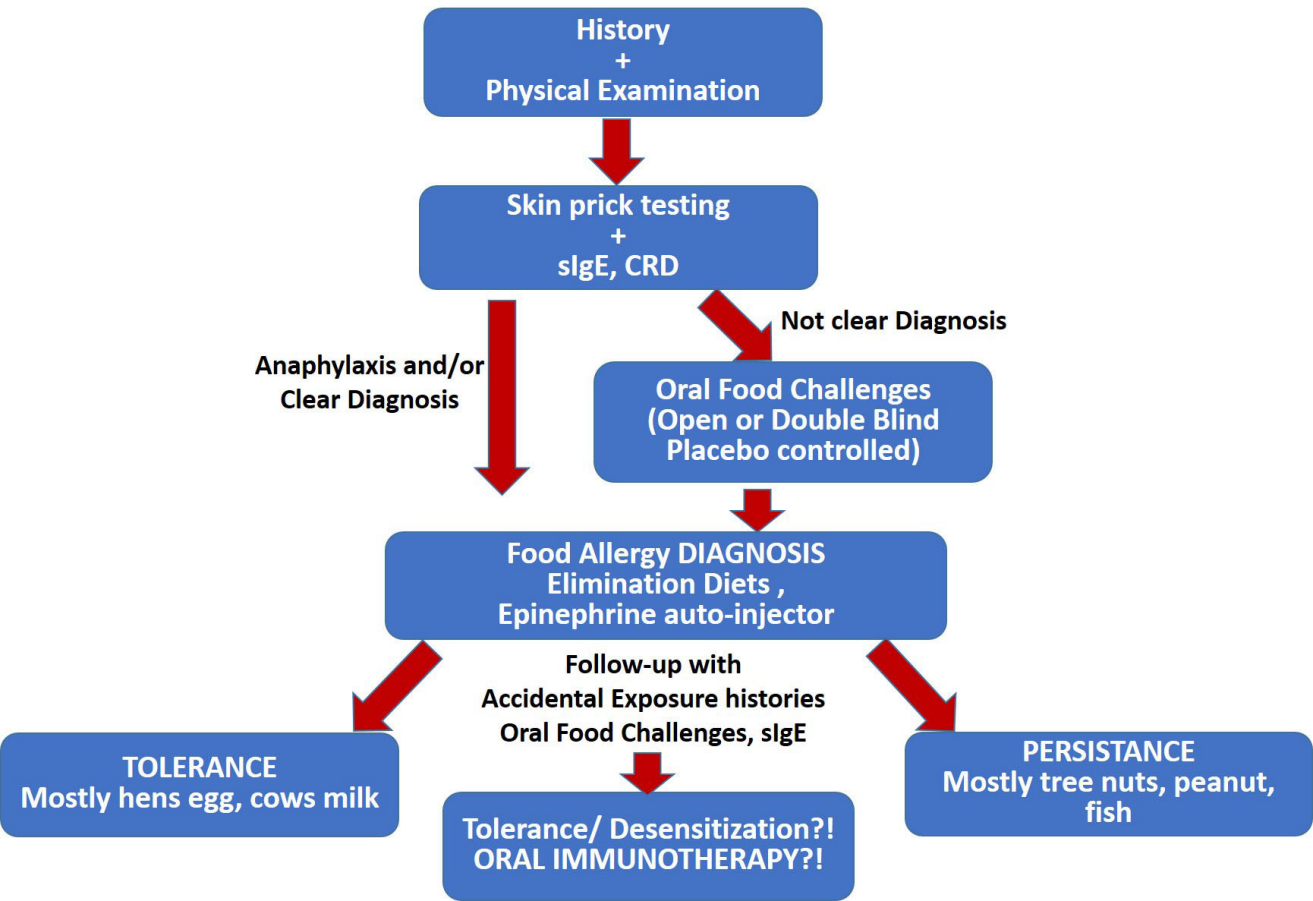
Practical approach to the diagnosis and follow-up of FA.

A practical approach to diagnosis and follow-up of IgE-mediated FA is presented in Figure 2. 

## 6. Prevention of food allergy in high-risk children

The study of Du Toit et al. [65] was the most informative research to understand the prevention approach for FA. High-risk infants (4–11 months old) with FA, who had severe eczema, egg allergy, or both, were included in the study. Patients were randomized into 2 groups, as the avoidance group and consumption group. The infants in the avoidance group avoided peanuts until the age of 5 years old, whereas infants in the consumption group started consuming peanuts at around 6 months of age. At the age of 60 months, the prevalence of peanut allergy was reported as 13.7% and 1.9% in the avoidance and consumption groups (P = 0.004), respectively. According to their study, the early introduction of peanuts to high-risk infants was suggested to prevent peanut allergy. In contrast, studies that have investigated the effects of early egg exposure in infants have reported inconsistent results for the prevention of egg allergy [21]. Oniwaza et al. [66] reported that delayed introduction to cow’s milk could be associated with IgE-mediated cow’s milk allergy. Urashima et al. [67] demonstrated that avoiding cow’s milk formula for at least first 3 days of life could prevent sensitization to cow’s milk. In addition, the restriction of allergenic foods during pregnancy or breast-feeding was not recommended to prevent FA [68]. 

## 7. Adult onset allergy to common foods

Cow’s milk allergy that persists into adulthood is uncommon, and most children with egg allergies also develop tolerance as they grow older [69]. On the other hand, the majority of peanut allergic adults acquire it in the childhood [69]. Adult-onset milk, egg, and peanut allergy may be rarely observed.

The underlying mechanism of FA in adults is primary sensitization to a nonfood allergen that has antigenic similarity to food, and is a cross-reactive type that results in the loss of tolerance to previously consumed foods (class 2 FA) [70]. Although less common, sensitization may occur directly to the food allergen (class 1 FA). IgE-mediated FA in adults may present with anaphylaxis, pollen FA syndrome (PFAS), oral allergy syndrome (OAS), alpha-gal allergy, or food-dependent exercise-induced anaphylaxis (FDEIA) [70].

Seafood allergy is one of the most common types of adult FA. Sensitization occurs upon consumption, skin contact, or inhalation of aerosolized allergens while cooking or food processing [71]. Tropomyosin and arginine kinase are the allergens responsible for cross-reactivity of shellfish with parasites, mites, and insects. However, component-resolved diagnosis will allow the identification of shellfish-specific allergens in the near future [71]. Parvalbumin is the major fish allergen that varies among species. Herring, codfish, salmon, and pollock were reported to be the most allergenic and cross-reacting, whereas mackerel, tuna, and halibut were reported as the least allergenic species [72]. Anisakis is a parasite that may contaminate fish, and cause allergic sensitization and the misdiagnosis of fish allergy. Scombroid poisoning is another type of reaction that mimics an allergic reaction, which results from the ingestion of improperly processed or stored fish that contain a high level of histamine. Although the reaction is clinically typical for IgE-mediated type allergens, it is non-IgE–mediated and is not reproducible.

## 8. Evaluation and management of food allergy in adults

After consumption of the offending food, the typical symptoms of IgE-mediated FA usually develop in 1 to 2 h. The symptoms range from mild urticaria/angioedema and gastrointestinal symptoms to severe anaphylaxis, and even death. The diagnosis of FA in adults should involve a stepwise approach. A detailed history of the reaction, and a list of all of the possible allergens ingested at least 6 to 8 h prior to reaction should be documented. A food that cross-reacts with an inhalant allergen (latex, pollen, or house dust mites) may be responsible for the current sensitization. Reaction history upon ingestion of cross-reacting foods with those inhalant allergens should be questioned. 

Diagnosis and treatment are similar to childhood cases; however, most of the food allergies in the adult population persist throughout life. 

## 9. Pollen food allergy syndrome

Instead of the commonly used term OAS, the term PFAS has better characterized the pathogenesis since its introduction in 1995 [73]. PFAS is defined as the development of allergic symptoms after the ingestion of fruits or vegetables in patients with pollen allergy-associated rhino conjunctivitis. Due to wide geographic variability, the true prevalence of the syndrome is difficult to determine. However, as 47%–70% of patients allergic to pollen have PFAS, the prevalence should range between 9.4% and 35% in the general population [73].

Plant food allergens belong to 3 protein superfamily classes, namely prolamin, cupin and pathogenesis-related (PR) proteins. Lipid transfer proteins (LTPs) belong to a prolamin superfamily that are both heat- and digestion-resistant (e.g., Mal d 3 and Pru p 3), and are found in many vegetables and fruits as pan-allergens [73]. The 2S albumins are also members of prolamin superfamily; however, their role in PFAS is limited. There is no described 2S albumin aeroallergen, so any identified 2S albumin allergen (e.g., Ana o 3 and Ara h 2) is a food allergen with sensitization that occurred through the gastrointestinal tract (class 1 FA), not as a cross-reactivity. Similar to 2S albumin, no cross-reacting aeroallergens have been found in the cupin superfamily (class 1 FA), and seed storage proteins (7S and 11S) are mostly related to allergy in the cupin superfamily of proteins [73]. PR-10 proteins are the most studied of the PR family. These proteins often denature with processing or cooking. Profilin family proteins are easily degraded in the stomach; therefore, systemic symptoms beyond oral symptoms are rare. However, due to the extensive homology among themselves, profilin sensitization occurs with multiple pollen-associated FA [73]. Table 1 shows the protein components of foods and pollens. 

The most common cross-reacting foods to profilins are melon, watermelon, tomato, banana, and citrus. The reactions are usually mild; nevertheless, more severe reactions have been reported as the result of cosensitization to LTPs and profilins [74]. PR-10 protein and Bet v 1 are responsible for the symptoms of individuals with birch pollen allergy upon the ingestion of apples, hazelnuts, carrots, and celery. LTPs are abundant, especially in the peels of Rosaceae fruit (pears and apples), and apricots, peaches, cherries, and plums. In the case of LTP sensitization, there is an increased risk of more severe reactions, and LTPs may cause FA in the absence of pollen allergy [74]. The severity of the reaction depends on the sensitization pattern, and whether it is to a stable (LTP) or labile (PR-10, profilin) protein. 

Most patients with PFAS initially exhibit oral symptoms, such as lip and mouth itching and/or angioedema with allergen exposure that will progress to systemic symptoms and even anaphylaxis with further exposure to the offending food. The misclassification of those patients as having simple oral allergy may lead to underdiagnosis and undertreatment [75]. Symptoms restricted to the oral cavity and lips are usually self-limited and do not progress with time; however, in a small number of patients, this reaction may progress to a more serious systemic type [75].

PFAS management includes the avoidance of the offending foods and provision of well-cooked or canned foods to patients. If there is uncertainty about the tolerance to known cross-reacting foods, challenge tests should be performed. The decision on the prescription of an adrenaline autoinjector should be carefully made considering the risk of systemic reaction and LTP sensitization. Patients with any form of systemic reaction should be prescribed an adrenaline autoinjector as a precaution [74].

## 10. Alpha-gal allergy

There are 3 distinct forms of red meat allergy, which are primary, pork-cat syndrome, and alpha-gal syndrome [76]. Primary beef allergy typically presents in childhood, pork-cat syndrome is most common in adolescents, whereas alpha-gal syndrome may present at any age [76].

Galactose- alpha-1,3-galactose (alpha-gal) is a newly identified food allergen. Reactions to this allergen occur in 2 forms, comprising delayed reactions after the ingestion of beef, pork, or lamb products, and immediate reactions after cetuximab exposure [77]. 

In 2009, it was shown in a group of patients that after eating mammalian meat, they experienced delayed anaphylaxis or urticaria/angioedema with a lack of immediate oral symptoms, and demonstrated IgE antibodies to alpha-gal [78]. There are alpha-gal epitopes within the saliva of ticks, and it is certain that sensitization to alpha-gal is related to the bites of hard ticks [76]. 

Contrary to other types of FA, even with high titers of sIgE to alpha-gal, the earliest symptom onset is 150 min (120 to 750 min) on average [76]. This raises the suspicion on the non-IgE mechanism; however, all evidence supports the fact that alpha-gal syndrome is an IgE-mediated disease.

A study including 261 patients allergic to meat (35 children, 226 adults), admitted to the University of Virginia Allergy Clinic, reported that the serum sIgE to alpha-gal was >0.35 IU/mL in 94% of the individuals, and when compared to the adults, there was male preponderance among the children (74% vs. 42% males), and the meat allergy was due to alpha-gal syndrome in 95% of these patients. It was observed that the presence of blood group B was protective against the development of the syndrome, and the syndrome was not associated with other atopic diseases [79]. 

Alpha-gal hypersensitivity is not only associated with food-related symptoms, but also other exposure, including gelatin or porcine/bovine-containing bioprosthetic heart valves, medications (e.g., heparin), and vaccines, can elucidate symptoms [80]. However, the amount of alpha-gal that is present in certain medications and their safety in patients with alpha-gal syndrome is currently unknown [81]. 

The management of alpha-gal syndrome includes dietary counseling for avoidance and a prescription for an adrenalin autoinjector. The titers of sIgEto alpha-gal may decrease over time, and the reintroduction of red meat into the diet may be possible when proof of tolerance has been established over several years [76]. 

## 11. Food-dependent exercise-induced anaphylaxis

Exercise-induced anaphylaxis can occur in 2 forms: anaphylaxis caused exclusively by exercise and that which occurs after eating and exercising, i.e. FDEIA. If anaphylaxis occurs after ingestion of a certain food that the patient is sensitized to, this is known as specific FDEIA, while if anaphylaxis occurs with any type of food, then it is called nonspecific FDEIA [82]. The true prevalence is unknown because in most cases, the sensitized food cannot be identified and patients are categorized as idiopathic anaphylaxis, or there is a lack of awareness among physicians.

The underlying mechanism of FDEIA is IgE-mediated FA that is aggravated by cofactors, such as exercise, nonsteroid antiinflammatory drugs, or alcohol. The symptoms, which may begin at any stage of exercise or just after exercise, may be aggravated with another cofactor, and may be unpredictable [82]. The responsible food is usually ingested within 4 h preceding exercise or after exercise [83]. Reaction starts as a sudden feeling of fatigue, flushing, and pruritus w/wo urticaria. Maintaining exercise may lead to severe anaphylaxis with hypotension and collapse. On the other hand, if patients stop exercising, the symptoms usually resolve [82].

Depending on the region and dietary habits, culprit foods may change and almost any food or combination of food allergens can cause FDEIA. Diagnosis is not easy, and requires a detailed clinical history and a high level of suspicion. The suggested criteria for diagnosis are [83]:

· Diagnosis of anaphylaxis during (within 1 h) exercise that occurs only if preceded by food ingestion.

· No other situation that can explain the clinical presentation.

In the case of the presence of a specific food trigger:

· The demonstration of sIgE to that food, either by skin or serum testing

· Patients usually consume the specific food safely without exercise, or safely exercise without consuming the specific food (in the absence of cofactors).

Skin testing, and/or in vitro sIgE testing, and if inconclusive, skin testing with fresh food is performed to show sensitization. A positive food + exercise testing confirms FDEIA diagnosis; however, a negative test does not always exclude diagnosis [83]. The identification and avoidance of contributing factors and foods is vital. Patients should carry adrenaline autoinjectors, stop exercise immediately if any symptoms occur, avoid the culprit food 4–6 h (at least 2 h) before exercise, not exercise alone, and preferably, exercise with an informed individual [83].

## 12. Future perspectives

The true prevalence of FA, especially among the adult population, is currently unknown, as self-reported and proven FA rates significantly differ. Specific IgE measurement has low specificity and may increase the overdiagnosis rates, and in most cases, food challenge is not performed. Highly cross-reactive carbohydrate epitopes and cross-contamination with other allergens contribute to lower specificity of the allergen extracts, both for SPT and sIgE measurement. 

Many proteins can be probed simultaneously and epitope pattern analysis can be performed with microarray-based assays using very small amounts of patient serum. However, more studies on the clinical application of the method should be performed. 

The basophil activation test (BAT), with a specific antigen, has shown that the biologic response and specificity of the test is probably higher than an sIgE measurement. However, the method is not widely available, as fresh serum is needed, and more clinical research investigating the role of BAT in FA should be conducted.

Greater microbial diversity in the gut may favor tolerance induction to foods. Mouse studies have shown that therapy with protective clostridial species has suppressed FA, and gut microbiota dysbiosis would be a potential future target for therapy [84]. However, well-designed prospective studies on humans are needed to understand if microbial changes or dysbiosis in the gut predispose an individual to the development of FA, and if so, what strategies could be used to induce tolerance. For FA prevention and treatment, diet manipulation, pro- and presynbiotic supplementation, and fecal microbiota transfer may be potential future research topics. 

## Conflict of interest

The authors declare no conflicts of interest.

## References

[ref1] (2010). Guidelines for the diagnosis and management of food allergy in the United States: report of the NIAID-sponsored expert panel. Journal of Allergy and Clinical Immunology.

[ref2] (2017). Food allergy: what we know now. The American Journal of the Medical Sciences.

[ref3] (2016). Food allergy: immune mechanisms, diagnosis and immunotherapy. Nature Review of Immunology.

[ref4] (2011). Prevalence of challenge-proven IgE-mediated food allergy using population-based sampling and predetermined challenge criteria in infants. Journal of Allergy and Clinical Immunology.

[ref5] (2017). The prevalence of food allergy and other allergic diseases in early childhood in a population-based study: health nuts age 4-year follow-up. Journal of Allergy and Clinical Immunology.

[ref6] (2018). Prevalence of clinic-defined food allergy in early adolescence: the school nuts study. Journal of Allergy and Clinical Immunology.

[ref7] (2011). The prevalence of food allergy in infants in Chongqing, China. Pediatric Allergy and Immunology.

[ref8] (2018). The public health impact of parent-reported childhood food allergies in the United States. Pediatrics.

[ref9] (2005). and main characteristics of schoolchildren diagnosed with food allergies in France. Clinical & Experimental Allergy.

[ref10] (2011). Phenotypes of IgE-mediated food allergy in Turkish children. Allergy & Asthma Proceedings.

[ref11] (2020). Immunoglobulin E-mediated food allergies differ in East Mediterranean children aged 0-2 years.

[ref12] (2013). Prevalence of confirmed IgE-mediated food allergy among adolescents in Turkey. Pediatric Allergy Immunology.

[ref13] (2013). Similar prevalence, different spectrum: IgE-mediated food allergy among Turkish adolescents. Allergologia et Immunopathologia.

[ref14] (2020). Allergy in Adults: presentations, evaluation, and treatment. Medical Clinics of North America.

[ref15] (2001). Prevalence of self-reported food allergy in U. Allergy & Asthma Proceedings.

[ref16] (2014). Prevalence of common food allergies in Europe: a systematic review and meta-analysis. Allergy.

[ref17] (2014). The epidemiology of food allergy in Europe: a systematic review and meta-analysis. Allergy.

[ref18] (2005). Food hypersensitivity in patients with seasonal rhinitis in Ankara. Allergologia et Immunopathologia.

[ref19] (2008). Confirmed prevalence of food allergy and non-allergic food hypersensitivity in a Mediterranean population. Clinical & Experimental Allergy.

[ref20] (2012). Standardizing double-blind, placebo-controlled oral food challenges: American academy of allergy, asthma & immunology-european academy of allergy and clinical immunology PRACTALL consensus report. Journal of Allergy and Clinical Immunology.

[ref21] (2018). Food allergy: a review and update on epidemiology, pathogenesis, diagnosis, prevention, and management. Journal of Allergy and Clinical Immunology.

[ref22] (2008). Testing for IgG4 against foods is not recommended as a diagnostic tool: EAACI task force report. allergy.

[ref23] (2018). Unproven diagnostic tests for adverse reactions to foods. Journal of Allergy and Clinical Immunology in Practice.

[ref24] (2019). Diagnosis and management of food allergy. Pediatric Clinics of North America.

[ref25] (2014). EAACI food allergy and anaphylaxis guidelines: diagnosis and management of food allergy. Allergy.

[ref26] (2005). Food allergy--accurately identifying clinical reactivity. Allergy.

[ref27] (2020). IgE allergy diagnostics and other relevant tests in allergy, a world allergy organization position paper. World Allergy Organ Journal.

[ref28] (2013). Factors that predict the clinical reactivity and tolerance in children with cow’s milk allergy. Annals of Allergy, Asthma & Immunology.

[ref29] IgE tests: in vitro diagnosis.

[ref30] (2014). Food allergy: a practice parameter update-2014. Journal of Allergy and Clinical Immunology.

[ref31] (2000). Specificity of allergen skin testing in predicting positive open food challenges to milk, egg and peanut in children. Clinical & Experimantal Allergy.

[ref32] (2014). The diagnosis of food allergy: a systematic review and meta-analysis. Allergy.

[ref33] (2013). Utility of casein-specific IgE levels in predicting reactivity to baked milk. Journal of Allergy and Clinical Immunology.

[ref34] (2015). Native and denatured egg white protein IgE tests discriminate hen’s egg allergic from egg-tolerant children. Pediatric Allergy Immunology.

[ref35] (2015). Predictive values of component-specific IgE for the outcome of peanut and hazelnut food challenges in children. Allergy.

[ref36] (2015). Clinical reactivity to soy is best identified by component testing to Gly m 8. Journal of Allergy and Clinical Immunology in Practice.

[ref37] (2017). Ana o 3-specific IgE is a good predictor for clinically relevant cashew allergy in children. Allergy.

[ref38] (2017). Jug r 1 sensitization is important in walnut-allergic children and youth. Journal of Allergy and Clinical Immunology in Practice.

[ref39] (2014). Clinical reactivity to hazelnut may be better identified by component testing than traditional testing methods. Journal of Allergy and Clinical Immunology in Practice.

[ref40] (2016). Cor a 14, hazelnut-specific IgE, and SPT as a reliable tool in hazelnut allergy diagnosis in eastern mediterranean children. Journal of Allergy and Clinical Immunology in Practice.

[ref41] (2016). Measurement of specific IgE antibodies to Ses i 1 improves the diagnosis of sesame allergy. Clinical & Experimantal Allergy.

[ref42] (2016). Ito. Journal of Allergy and Clinical Immunology in Practice.

[ref43] (2015). Ara h 2 and Ara 6 are the best predictors of severe peanut allergy: a double-blind placebo-controlled study. Allergy.

[ref44] (2013). Sensitization to Cor a 9 and Cor a 14 is highly specific for a hazelnut allergy with objective symptoms in Dutch children and adults. Journal of Allergy and Clinical Immunology.

[ref45] (2020). Conducting an oral food challenge: an update to the 2009 adverse reactions to foods committee work group report. Journal of Allergy and Clinical Immunology in Practice.

[ref46] (2017). Committee for Japanese pediatric guideline for food allergy TJSoPA, Clinical Immunology TJSoA. Japanese guidelines for food allergy 2017. Allergology International.

[ref47] (2010). When is an oral food challenge positive. Allergy.

[ref48] (2014). The natural history of egg allergy in an observational cohort. Journal of Allergy and Clinical Immunology.

[ref49] (2007). The natural history of egg allergy. Journal of Allergy and Clinical Immunology.

[ref50] (2013). The natural history of milk allergy in an observational cohort. Journal of Allergy and Clinical Immunology.

[ref51] (2007). The natural history of IgE-mediated cow’s milk allergy. Journal of Allergy and Clinical Immunology.

[ref52] (2009). The natural history of wheat allergy. Annals of Allergy Asthma & Immunology.

[ref53] (2015). Natural history of peanut allergy and predictors of resolution in the first 4 years of life: a population-based assessment. Journal of Allergy and Clinical.

[ref54] (2005). The natural history of tree nut allergy. Journal of Allergy and Clinical Immunology.

[ref55] (2013). World allergy organization anaphylaxis guidelines: 2013 update of the evidence base. International Archives of Allergy and Immunology.

[ref56] (2017). Food allergy. New England Journal of Medicine.

[ref57] (2012). Oral immunotherapy for treatment of egg allergy in children. New England Journal of Medicine.

[ref58] (2015). Sublingual immunotherapy for peanut allergy: long-term follow-up of a randomized multicenter trial. Journal of Allergy and Clinical Immunology.

[ref59] (2008). Sublingual immunotherapy for hazelnut food allergy: a follow-up study. Annals of Allergy Asthma and Immunology.

[ref60] (2010). Cow’s milk epicutaneous immunotherapy in children: a pilot trial of safety, acceptability, and impact on allergic reactivity. Journal of Allergy and Clinical Immunology.

[ref61] (2016). Food allergen immunotherapy: current status and prospects for the future. Journal of Allergy and Clinical Immunology.

[ref62] (2009). Safety of a peanut oral immunotherapy protocol in children with peanut allergy. Journal of Allergy and Clinical Immunology.

[ref63] (2013). Safety and predictors of adverse events during oral immunotherapy for milk allergy: severity of reaction at oral challenge, specific IgE and prick test. Clinical and Experimental Allergy.

[ref64] (2017). Omalizumab facilitates rapid oral desensitization for peanut allergy. Journal of Allergy and Clinical Immunology.

[ref65] (2015). Randomized trial of peanut consumption in infants at risk for peanut allergy. New England Journal of Medicine.

[ref66] (2016). The Association of the delayed introduction of cow’s milk with IgE-mediated cow’s milk allergies. Journal of Allergy and Clinical Immunology in Practice.

[ref67] (2019). Primary prevention of cow’s milk sensitization and food allergy by avoiding aupplementation with cow’s milk formula at birth: a randomized clinical trial. JAMA Pediatrics.

[ref68] (2013). Primary prevention of allergic disease through nutritional interventions. Journal of Allergy and Clinical Immunology in Practice.

[ref69] (2009). Food allergy in adolescents and adults. International Medicine Journal.

[ref70] (2017). Adult-onset food allergies. Annals of Allergy Asthma and Immunology.

[ref71] (2020). Clinical management of aeafood allergy. Journal of Allergy and Clinical Immunology in Practice.

[ref72] (2005). Allergy to fish parvalbumins: studies on the cross-reactivity of allergens from 9 commonly consumed fish. Journal of Allergy and Clinical Immunolgy.

[ref73] (2019). Pollen food allergy syndrome (PFAS): a review of current available literature. Annals of Allergy Asthma and Immunology.

[ref74] (2016). Should patients with pollen fruit syndrome be prescribed an automatic epinephrine injector?. Current Opinionin Allergy and Clinical Immunology.

[ref75] (2015). Management of pollen food and oral allergy syndrome by health care professionals in the United Kingdom. Annals of Allergy Asthma and Immunology.

[ref76] (2019). Red meat allergy in children and adults. Current Opinion in Allergy and Clinical Immunology.

[ref77] (2018). Predictive values of alpha-gal IgE levels and alpha-gal IgE: total IgE ratio and oral food challenge-proven meat allergy in a population with a high prevalence of reported red meat allergy. Pediatric Allergy Immunology.

[ref78] (2009). Delayed anaphylaxis, angioedema, or urticaria after consumption of red meat in patients with IgE antibodies specific for galactose-alpha-1,3-galactose. Journal of Allergy and Clinical Immunology.

[ref79] (2019). Investigation into the alpha-Gal syndrome: characteristics of 261 children and adults reporting red meat allergy. Journal of Allergy and Clinical Immunology in Practice.

[ref80] (2019). Successful intravenous heparin administration during coronary revascularization surgery in a patient with alpha-gal anaphylaxis history. Annals of Allergy Asthma and Immunology.

[ref81] (2019). Tolerance of porcine pancre-tic enzymes despite positive skin testing in alpha-gal allergy. Journal of Allergy and Clinical Immunology in Practice 2019. doi: 10.

[ref82] (2019). Diagnosis and prevention of food-dependent exercise-induced anaphylaxis. Expert Review of Clinical Immunology.

[ref83] (2017). Food-dependent, exercise-induced anaphylaxis: diagnosis and management in the outpatient setting. Journal of Allergy and Clinical Immunology in Practice.

[ref84] (2019). Microbiota therapy acts via a regulatory t cell, my D88/ROR gammat pathway to suppress food allergy. Nature Medicine.

